# Molecular Characterization of Hard Ticks Infesting Camels in the Northern Region of Saudi Arabia Using the Barcoding Gene, Mitochondrial *Cytochrome oxidase subunit I*

**DOI:** 10.3390/life13071535

**Published:** 2023-07-10

**Authors:** Fevzi Bardakci, Sarah Hilan Mohammed Al-Subaie, Riadh Badraoui, Mohd Adnan, Arif Jamal Siddiqui

**Affiliations:** 1 Department of Biology, College of Science, University of Ha’il, Ha’il P.O. Box 2440, Saudi Arabia; s20200091@uoh.edu.sa (S.H.M.A.-S.); mo.adnan@uoh.edu.sa (M.A.); ar.siddiqui@uoh.edu.sa (A.J.S.); 2 Section of Histology-Cytology, Medicine Faculty of Tunis, University of Tunis El Manar, Tunis 1007, Tunisia; riadh.badraouir@fmt.utm.tn

**Keywords:** hard ticks, *cytochrome oxidase subunit I*, *COI*, phylogenetics, population genetics, *Hyalloma*, acari, ixodidae

## Abstract

The present study aimed to molecularly identify and characterize the hard ticks infesting camels from the northern region (Ha’il province) of Saudi Arabia using the mitochondrial barcoding gene *cytochrome oxidase subunit I* (*COI*). The sequences of tick samples from camels in three regions of Ha’il were aligned with those previously reported from different geographic regions, revealing nine haplotypes, of which six were newly described in this study for the first time. These haplotypes were used to determine their phylogenetic relationships using the maximum likelihood method, displaying two distinct clades corresponding to *Hyalomma dromedarii* and *H. impeltatum*. Moreover, the haplotypes showing the highest homology with those deposited in NCBI-GenBank from different geographic regions, including Saudi Arabia, were obtained and combined to determine their phylogenetic relationships among them. The results showed that the haplotypes belonging to two clades were grouped with those previously determined as *H. dromedarii* and *H. impeltatum*. Moreover, the presence of *H. scupense* (syn. *H. detritum*) together with *H. impeltatum* suggests possible asymmetrical hybridization and mitochondrial introgression between these species. *H. scupense* infesting different mammal species apart from camels were also clustered in a different clade, indicating the presence of different lineages of this species that show different host specificities.

## 1. Introduction

Hard ticks are obligate hematophagous ectoparasitic arachnids that infest reptiles (snakes, iguanas, and lizards), amphibians, birds, marsupials, and placental mammals [1]. Ticks have significant economic impacts as they serve as vectors of a variety of infectious diseases of great interest to humans, livestock, and wild animals worldwide [2,3,4]. To date, a total of 55 tick species have been identified from domestic animals in the Middle East and North Africa (MENA) of which the soft ticks (Argasidae) are represented by two genera (*Otobius* and *Ornithodoros*), while hard ticks (Ixodidae) are represented by six genera (*Hyalomma*, *Haemaphysalis*, *Amblyomma*, *Ixodes*, *Rhipicephalus*, and *Dermacentor*. The hard tick genus *Hyalomma* has an estimated 30 species and subspecies in Africa (Afrotropical), Asia (Oriental), and Europe (Palaearctic). *Hyalomma* ticks have been a great concern to humans as they are reservoirs of various infectious agents that infest humans, livestock, and wild animals. The most common tick species in the MENA region is *Hyalomma dromedarii* due to large-scale camel farming. Therefore, the majority of research has been carried out on hard ticks in the MENA region [5].

A literature review on the fauna of tick species has shown that *Hyalomma*, *Amblyomma*, *Haemaphysalis* and *Rhipicephalus* are the major tick genera in Saudi Arabia [5,6,7,8,9,10,11,12]. A total of 37 tick species and subspecies infesting livestock and wild animals have been reported from Saudi Arabia [6], including 15 ixodid species and subspecies (*R. kohlsi*, *H. dromedarii*, *H. sulcata*, *H. excavatum*, *H. anatolicum*, *H. arabicum*, *H. rufipes*, *H. impeltatum*, *H. schulzei*, *H. turanicum*, *R. camicasi*, *R. turanicus*, *R. praetextatus*, *A. variegatum*, and *A. gemma*) and one aragasid species (*Argas persicus*) [13]. Of these ticks, *H. dromedarii*, *H. schulzei*, and *H. asiaticum* are the species most closely related to and associated with camels. The most prevalent tick species in Saudi Arabia is *H. dromedarii* mainly due to large-scale camel farming [5,14].

Dromedary camels (*Camelus dromedaries*) are of great importance in the Arabian Peninsula, including in Saudi Arabia, mainly as a source of meat and milk production [12]. The infestation of livestock, including camels, has been reported in previous studies [13,14,15]. Due to the importance of the ticks mentioned above, the identification of tick species by geographic distribution and their geographic lineages is very important for the control and prevention of tick-mediated infectious diseases as it is difficult to distinguish between species with morphological similarities such as *H. dromedarii* and *H. truncatum* [16].

The identification and phylogeny of species and subspecies of ticks are of great concern due to their host preferences that are important in the prevention and control of tick-mediated infectious diseases. The characterization of ticks solely based on morphological characteristics is not sufficient for the identification of some tick species. Therefore, combined morphological characteristics and molecular markers of different tick species provide more robust information on their classification and phylogeny [17,18,19,20]. A study based on mitochondrial (mtDNA) and nuclear DNA (nDNA) sequences of *H. dromedarii*, *H. truncatum*, and *H. marginatum rufipes* ticks from one-humped camels in Ethiopia has shown some discrepancies between morphology and mtDNA phylogeny due to the gene flow among these species, which was not reflected in their morphology [18]. A few studies on the population genetics and phylogeny of the most common tick species in Saudi Arabia (*H. schulzei*, *H. dromedarii*, *H. anatolicum*, *H. impeltatum*, *Boophilus kohlsi*, and *Rhipicephalus turanicus*) have been carried out due to their economic and veterinary importance. The two most common tick species infesting camels and cattle from the Ha’il region of Saudi Arabia have been identified using protein electrophoresis [21]. Fallatah et al. [22] used the barcoding gene mtDNA *COI* for the phylogenetic analysis of *H. dromedarii* from the eastern region of Saudi Arabia and found that *H. dromedarii* is monophyletic with two distinguished lineages. In another study [23], both morphological characteristic and barcoding gene mtDNA *COI* sequence analyses were used for the identification of ticks infesting camels and cattle in Hofuf in eastern Saudi Arabia. The molecular characterization of these ticks has proved the presence of two species (*H. dromedarii* and *H. anatolicum*), confirming their morphological description.

The present study aimed to use the barcoding gene mitochondrial *Cytochrome oxidase c subunit I* (*COI*) for the molecular characterization and phylogeny of hard ticks from camels in the Ha’il Province of Saudi Arabia. Findings from this project might open a new window in the development of a molecular identification tool for possible tick strains in the northern region of Saudi Arabia for the prevention and control of tick-related diseases as well as their interactions with their hosts.

## 2. Materials and Methods

**Figure 1:**
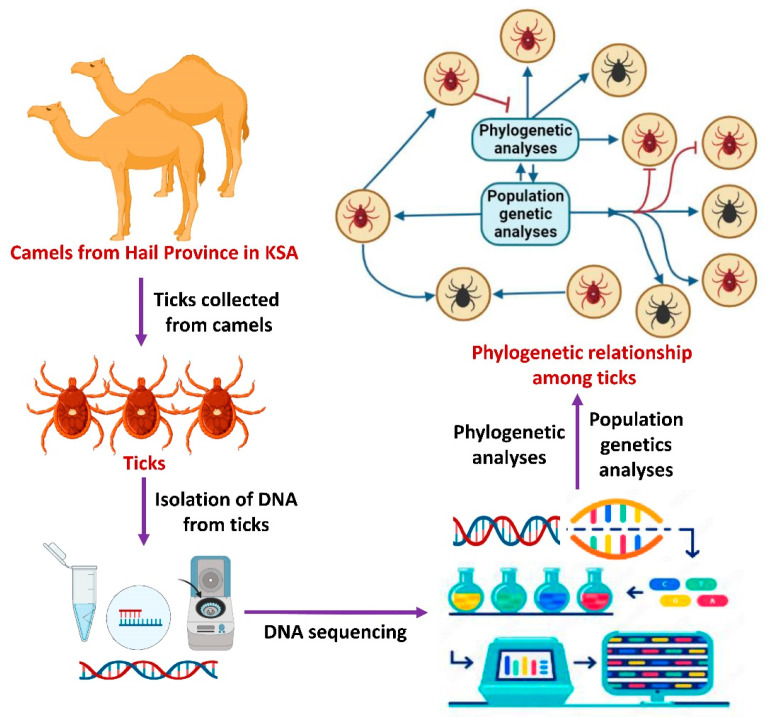
A summary of the methods followed in this study.

Our methodology from the collection of tick samples to the analyses of the sequence data used in this study is summarized in Figure 1.

### 2.1. Study Area and Sample Collection

Tick samples were collected from different locations in the Ha’il region. The Ha’il Province is located in the middle of the northern central part of Saudi Arabia, and the city of Ha’il is considered the fifth largest city in the kingdom of Saudi Arabia, covering an area of 118,322 sqm, which extends from 25°29′ N to 38°42′ E. The Ha’il province has an altitude that varies from 900 to 1350 m.

The climate in the Ha’il region is generally arid and extra arid, with hot summers and cold winters and highs of 45 to 27 °C and lows of 23 to 3 °C [24]. With little to no precipitation in June, July, August, and September, the Ha’il region experiences yearly rainfall averaging between 100 and 120 mm. The region experiences sporadic and unpredictable rainfall; most falls during the winter and early fall, with the highest daily precipitation occurring in November (32.0 mm/day) and an average yearly rainfall of 104.4 mm/day [25]. However, no rain has been recorded during the summer months.

Tick samples (100 ticks: 4 to 5 specimens per animal) were collected from twenty healthy, naturally infested camels in the Ha’il province regions of Al Qaid (27°50′27″ N 41°53′24″ E), Al Khotha (27°59′57″ N 41°44′12″ E), and Al Ajfar (27°28′22″ N 42°59′40″ E); placed individually in 1.5 mL microcentrifuge tubes containing absolute ethanol; and stored at +4 °C until used in the experiments. Tick samples were identified using a stereomicroscope based on morphology, according to Dantas-Torres [26] and Barker and Walker [27]. PCR and sequencing assays were successful in 50 of the 100 tick samples, including 18 from Al Qaid, 14 from Al Khotha, and 18 from Al Ajfar.

### 2.2. DNA Isolation and Amplification of mtDNA COI Gene

An individual tick sample was shredded into pieces in a 1.5 microcentrifuge tube with scissors. A commercial DNA isolation kit Exgene™ Clinic SV kit (GeneAll Biotechnology Co., Ltd., Seoul, Republic of Korea) was used in this study following the manufacturer’s instructions. The resulting DNA sample was stored at −20 °C till used.

After DNA isolation, DNA amplification was performed by PCR using WizPure™ PCR 2X MasterMix (Wizbisolutions, Republic of Korea) containing DNA polymerase, MgCl_2_, dNTP, enhancers, and stabilizers. The mitochondrial *COI* barcoding gene was amplified from total tick genomic DNA using primer pair LCO1490: 5′-GGTCAACAAATCATAAAGATA-TTGG-3′ and HCO2198:

5′-TAAACTTCAGGGTGACCAAAAAATCA-3′, which have previously been used to successfully amplify this gene fragment in a variety of animals [28]. The total volume of 20 µL PCR reaction contained 1X PCR 2X MasterMix, 0.5 µM each forward and reverse primer, and 100 ng total genomic DNA.

The contents were thoroughly mixed by gentle pipetting, followed by brief centrifugation in a microcentrifuge tube, and placed in a thermocycler (Applied Biosystems™ Veriti Thermocycler, ABI, Waltham, MA, USA) set as follows to amplify the target regions of mtDNA selectively: initial denaturation at 95 °C for 4 min, 35 cycles of denaturation at 94 °C for 30 s, annealing at 65 °C for 1 min, extension at 72 °C for 30 s, and a final extension at 72 °C for 3 min after cycle 35.

The resulting PCR products were separated into 0.9% agarose gel (GeneDireX, Las Vegas, NV, USA) in 1x TBE (Tris-borate-EDTA) stained with GelGreen Nucleic Acid Gel Stain (Biotium, Inc., Hayward, CA, USA). The electrophoresis was conducted for 50 min at 70 volts. Gels were viewed and photographed using the Blook Led Transilluminator device (GeneDireX, Las Vegas, NV, USA).

### 2.3. DNA Sequencing

The PCR products were cleaned to prepare for DNA sequencing using ExoSAP-IT™ Express PCR Product Cleanup Reagent (Thermo Fisher Scientific, Waltham, MA, USA) according to the instructions. After the PCR clean-up process, DNA sequencing was carried out to sequence the PCR fragment of mtDNA *COI* using the “Big Dye Terminator v.3.1 Cycle Sequencing Ready Reaction” kit (Applied Biosystems, Waltham, MA, USA). Sequencing was performed in two directions for each amplified *COI* fragment using forward and reverse primers used for the PCR to obtain more reliable sequences. The Sanger dideoxy sequencing method [29] was performed in both directions using forward and reverse primers (Atlas Biotechnology Ankara, Ankara, Turkey). The DNA sequence analysis was performed on an ABI 3130XL using capillary electrophoresis. The resulting DNA sequence chromatograms were edited, assembled into a single contig, and aligned using BioEdit software version 7.0.9.0 [30]. As a result, mtDNA COI sequences were successfully obtained from 50 tick individuals belonging to three localities and used for further analyses.

### 2.4. Phylogenetic Analyses

The aligned sequences from tick samples from Ha’il province were analyzed to determine the number of mtDNA haplotypes using DnaSP v. 5.1 [31]. Each mtDNA *COI* haplotype sequence obtained from the tick samples was subjected to a BLAST search, which is implemented in the NCBI (BLASTN 2.7.1+) [32], to determine their sequence homology with respect to those from different geographical areas deposited in the NCBI database for their species identification and phylogenetic relationships. The sequences with the highest homologies as well as those belonging to *Nossoma monstrosum* as an outgroup were combined and aligned successfully with these sequences using the Clustal W software [33] in BioEdit version 7.1.3.0 [31]. As a result, 500 bp of mtDNA *COI* region was successfully aligned after trimming manually. In addition, all haplotypes identified to date were combined with those obtained from this study, aligned, and trimmed manually. Since many haplotypes reported previously have sequence numbers around 250 bp, sequences were aligned with software online (https://users-birc.au.dk/palle/php/fabox/alignment_trimmer.php accessed on 10 April 2023). Only 284 bp sequences remained after trimming their ends [22]. Therefore, alignment with the software online was not used in the final presentation of the results.

Maximum likelihood (ML) [34] methods were used to construct a phylogenetic tree for the combined data in MEGA ver. 11.0.13 software [35]. First, the evolutionary distances, measured in the number of base substitutions per site, were estimated using the Tamura–Nei model [36], which is the model that best fits our data, as inferred from MEGA 11.0.13 software. Then, for each sequence, all gaps and missing sites were eliminated using the pairwise deletion option for each sequence pair containing 500 sites altogether. Finally, the reliability of the branches of the phylogenetic trees was tested using 1000 bootstrap replicates. Furthermore, an ML tree was constructed from the combined sequences of mtDNA haplotypes to determine their phylogenetic relationships.

The genetic variation between the resultant mtDNA haplotypes was estimated via descriptive values, including the number of haplotypes (H), haplotype diversity (Hd), and nucleotide diversity [37], using the software package DnaSP v. 5.1 [31]. The evolutionary divergence between sequences of mtDNA haplotypes was estimated using the Tamura–Nei model [36] in MEGA 11.0.13 software 3.

## 3. Results

### 3.1. Sequence Analysis of Hard Ticks Based on mtDNA COI Sequences

**Figure 2:**
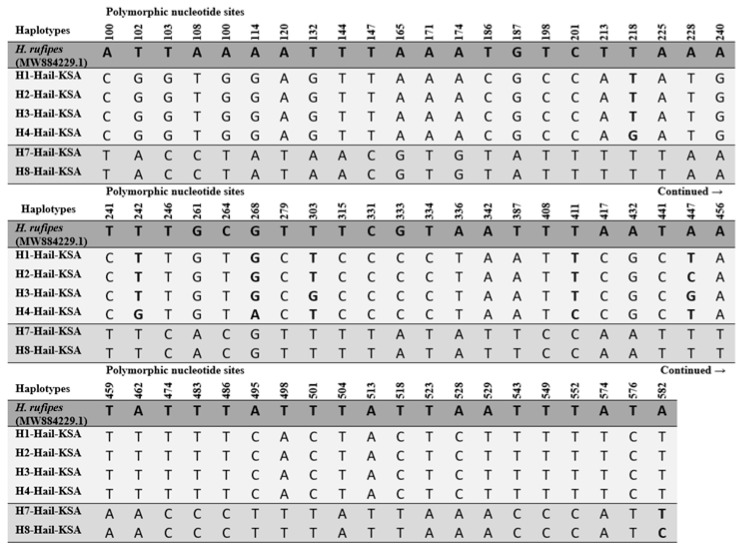
Polymorphic nucleotide sites of the novel mtDNA *COI* haplotypes in the tick samples from the Ha’il region aligned with *H. rufipes* mtDNA *COI* as a reference sequence. Polymorphic nucleotide sites within each haplogroup (Clade I: H1, H2, H3, and H4; Clade II: H7 and H8 in Figure 3) are given in bold.

The mtDNA barcoding gene, *COI*, is regarded as the most useful molecular marker to discriminate among animal species and determine their phylogenetic relationships. Therefore, the sequences of *COI* from 50 hard tick samples were aligned with the complete mtDNA *COI* sequences of *H. rufipes* as a reference to determine the positions of their polymorphic sites (Figure 2). As a result, 500 bp of a partial sequence of the mtDNA *COI* obtained after trimming their ends were composed of 64 variable sites that produced nine mtDNA haplotypes, of which six (H1, H2, H3, H4, H7, and H8) were newly described in this study. The sequences of the new *COI* haplotypes were deposited in the NCBI-GenBank with accession numbers OR050804-OR050809. The haplotype diversity and nucleotide diversity of all *COI* sequences from the hard ticks from the Ha’il region were 0.657 and 0.04954, respectively (Table 1).

The mtDNA *COI* haplotypes determined from the Ha’il region were assigned to the areas where the tick samples were collected (Table 2). The results showed that the haplotype H1 was the most common, with 14, 9, and 5 tick samples from Al Qaid, Al Khotha, and Al Ajfar, respectively. The second most common haplotype, H9, has only been determined in the tick samples from the Al Ajfar area. Moreover, each of the three tick samples from the Al Qaid region had one of the three specific haplotypes (H3, H4, or H5). Apart from these haplotypes specific to these regions, H6, H7, and H8 were only detected from the tick samples from the Al Khotha area.

Genetic variation between tick samples from the study areas was compared to determine the mtDNA *COI* diversity among them (Table 3). The results clearly showed that the Al Ajfar area has the highest nucleotide diversity in the mtDNA *COI*, while the lowest is found between Al Qaid and Al Khotha. Nucleotide and haplotype diversity among the ticks from the study areas put forward that Al Ajfar ticks are genetically different from those from the other two areas studied.

The haplotypes found in this study were combined with those reported from different geographic regions to estimate the evolutionary divergence between them (Table 4). All haplotypes were aligned, and their nucleotide polymorphisms were detected.

These results allowed us to determine whether any haplotypes from the Ha’il region are new or previously found in any other geographic region. As mentioned above, six haplotypes (H1, H2, H3, H4, H7, and H8) found in the Ha’il region were new for the hard tick samples. A pairwise comparison between all the haplotypes of the hard ticks showed that H7 and H8 differentiated from the other haplotypes found in the Ha’il region. Moreover, the pairwise comparison between all the haplotypes revealed the sequence similarity between these two haplotypes and previously described haplotypes including H5-Hdro KSA MK177459.1 and H4-Hdro KSA MK177458.1 [22].

### 3.2. Identification of Hard Ticks and Their Phylogenetic Relationships

**Figure 3:**
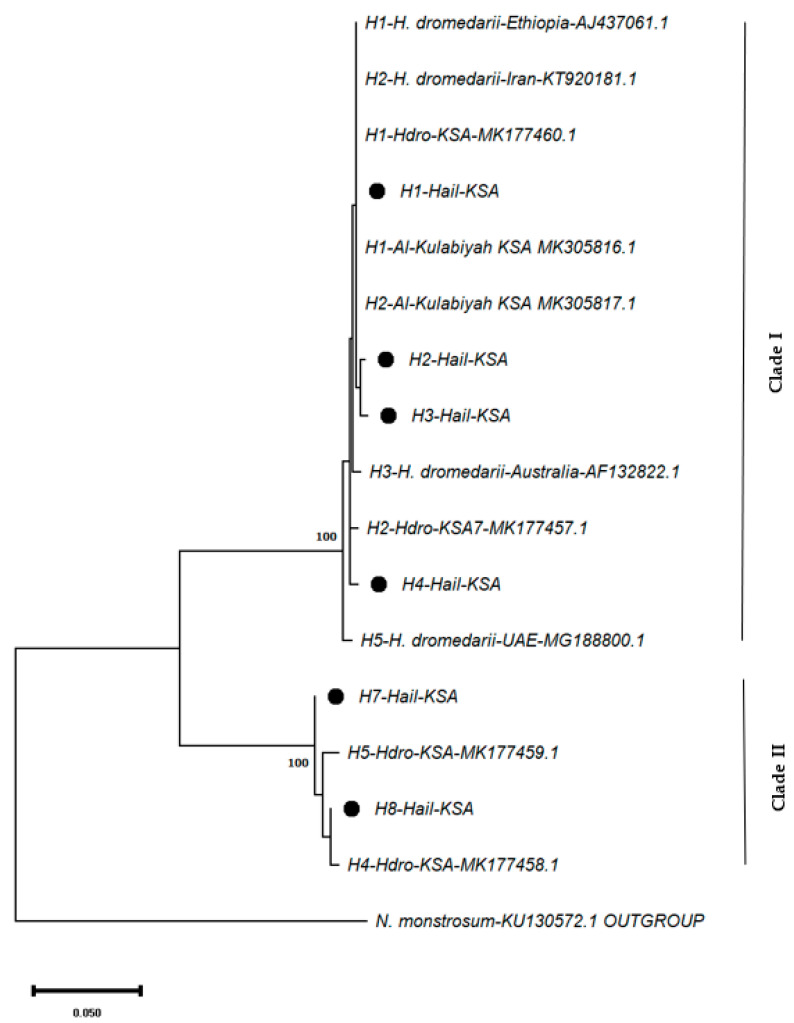
ML haplotype tree of the mtDNA *COI* haplotypes of the hard ticks infesting camels. The mtDNA *COI* haplotypes detected in tick samples in this study are denoted by the • symbol.

To determine the phylogenetic relationships of all mtDNA haplotypes, an ML tree was generated from the *COI* sequences of the hard ticks (Figure 3). The resulting tree displayed two clades. The haplotypes obtained from this study were also split between these clades: while H1, H2, H3, and H4 were placed in the first clade, the remaining ones, H7 and H8 were placed in the second clade. The haplotypes placed in Clade I belong to the tick samples from Al Qaid (H1, H2, H3, and H4), Al Khotha (H1 and H2), and Al Ajfar (H1), while those set in Clade II were from the ticks from Al Khotha (H7 and H8). These two clades are within *H. dromedarii* according to their clustering with the haplotypes previously defined for this hard tick species.

**Figure 4:**
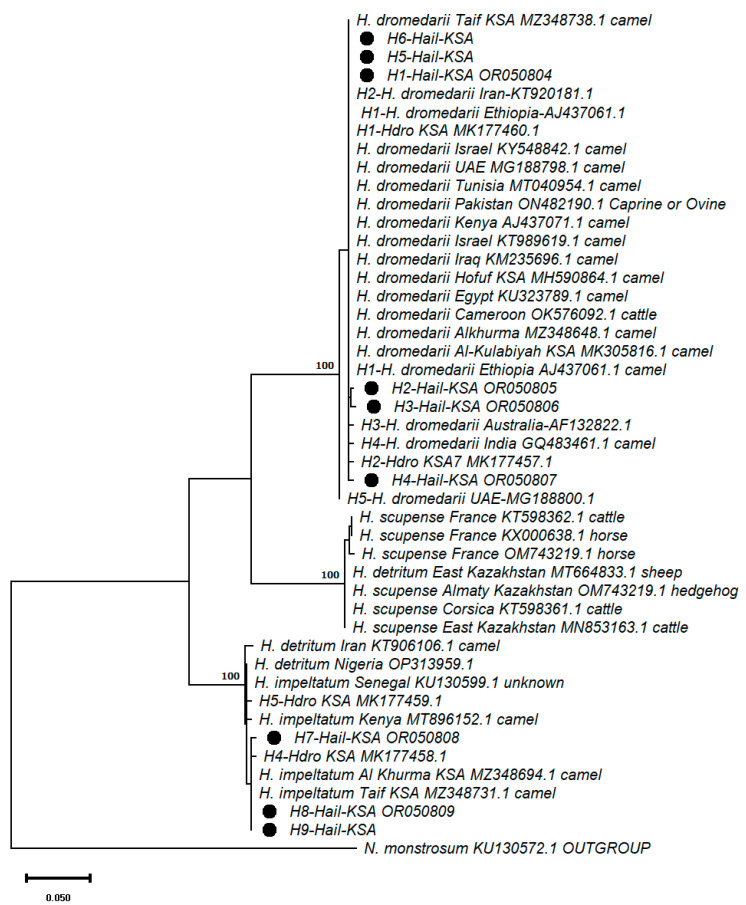
ML tree of the mtDNA *COI* sequences of the hard tick species from different geographical regions. The mtDNA *COI* haplotypes detected in tick samples in this investigation are denoted by the • symbol.

Further phylogenetic analyses of the *COI* sequences of hard tick populations from the different geographic regions (obtained from the NCBI-GenBank), including the samples from this study, were performed to confirm if these two clades belonged to *H. dromedarii* or different species. Therefore, the haplotypes determined from this study were subjected to a BLAST search implemented in the GenBank, and the sequences with the highest homologies were used to build an ML tree. In addition, *COI* sequences from the ticks infesting different hosts apart from the camels were also included in the dataset to determine the host specificity of the hard ticks. The ML tree clearly showed that Clade I and II haplotypes do not belong to the same tick species (Figure 4). The haplotypes in Clade I were clustered with the haplotypes previously defined from *H. dromedarii*. However, the haplotypes placed in Clade III were clustered with *H. scupense* and *H. impeltatum*. Moreover, some *COI* sequences displaying the higher homologies were not specific to camels, and their hosts were cattle, sheep, horses, and hedgehogs. Therefore, the sequences belonging to the ticks from these hosts were also separated into a different clade (Clade II), defined as *H. scupense*. These results revealed that *H. scupense* ticks infesting cattle, sheep, horses, and hedgehogs have genetically different lineages displaying host specificity.

## 4. Discussion

The present study aimed to molecularly identify and characterize the hard ticks infesting dromedary camels using the barcoding mtDNA *COI* sequences. The sequences of the *COI* were analyzed to determine a genetic variation within the ticks from Ha’il Province, Saudi Arabia. As a result, the analysis of the *COI* sequences revealed nine mtDNA haplotypes, of which six were newly described for the first time in this study. The estimates of evolutionary divergence between mtDNA *COI* haplotypes in camel hard ticks ranged from 0.02 to 0.136. The mtDNA *COI* haplotype and nucleotide diversity of hard tick samples from the Ha’il region were 0.657 and 0.04954, respectively. Haplotype and nucleotide diversity in *H. dromedarii* (Clade I) from the eastern part of Saudi Arabia, on the other hand, were 0.376 and 0.0018, respectively, and were lower than in Clade II (Haplotype diversity: 0.909 and nucleotide diversity: 0.00640) [22]. The increased haplotype and nucleotide diversity in Clade II suggests that the haplotypes within this clade may not belong to *H. dromedarii*, which will be examined more in this section. On average, these results were similar to those estimated in this study. mtDNA *COI* inter- and intraspecies divergences were 0.039 and 0.113, respectively, in seven *Hyalloma* species [38]. The genetic variability of *H. dromedarii* from the Taif province of Saudi Arabia assessed via ISSR markers showed a 0.56 intraspecific genetic similarity [39].

The phylogenetic analyses of these haplotypes and those reported in previous research clustered them into two clades: Clade I (including H1, H2, H3, and H4), clustered with the haplotypes H1-*Hdro*-KSA and H2-*Hdro*-KSA, and Clade II (including H7 and H8), clustered with H4-*Hdro*-KSA and H5-*Hdro*-KSA, as reported in Fallatah et al. [22] (Figure 3). Therefore, the results of this study are in accordance with those of Fallatah et al. [22]. In order to test if these two clades belong to *H. dromedarii*, the haplotypes obtained from this study were combined with *COI* sequences with the highest homology determined by the NCBI BLAST search. Phylogenetic analyses conducted by constructing the ML tree of these combined *COI* sequences showed that the haplotypes in Clade III do not match with *H. dromedarii* haplotypes but rather coincide with the sequences belonging to *H. scupense* and *H. impeltatum* (Figure 4). The fact that Fallatah et al. [22] only provided a haplotype tree and not a species tree led to their identification of Clade II haplotypes, H4-*Hdro*-KSA and H5-*Hdro*-KSA, as being those of *H. dromedarii* (Figure 3).

Although *H. scupense* seems to be within Clade II (Figure 4), having other mammalian hosts, two haplotypes of this species reported from camels in Iran and Nigeria clustered with Clade III were dominated by *H. impeltatum* haplotypes from different geographic regions, including Saudi Arabia. Two haplotypes reported in camels from Al Khurma (Al Khurma KSA MZ348694.1) and Taif (Taif KSA MZ348731.1) were placed in Clade III (Figure 4), indicating the presence of *H. impeltatum* in these regions. The results of a survey on *Hyalomma* ticks from 10 camels belonging to two farms in the Al-Qasim Province of Saudi Arabia showed that *H. impeltatum* is the second most common species after *H. dromedarii* [7]. *H. impeltatum* was also reported as having the third highest occurrence in camels in a study on the tick species infesting domestic and wild animals in Riyadh Province, Saudi Arabia. These findings suggest that haplotypes H6-H11 within Clade II, reported in Fallatah et al. [22], do not belong to the *H. dromedarii* species but to *H. impeltatum.* These haplotypes are not included in the phylogenetic analyses of camel ticks from camels since Fallatah et al. [22] do not provide the sequence accession number.

One of the key findings of this study is that the Al Ajfar region has the highest nucleotide diversity in the mtDNA *COI* compared to the Al Qaid and Al Khotha ticks. The Al-Ajfar region, located near the eastern border of Ha’il Province, is one of the largest agricultural areas in Saudi Arabia. It is clear that the main reason for separating this region from Al-Qaid and Al-Khotha is the high prevalence of haplotype 9, which is unique to ticks from this region.

The presence of *H. scupense* in Clade III might be due to the asymmetric hybridization between this species and *H. impeltatum*. The hybridization and co-occurrence of two tick species in the same habitat, suggesting the lack of genetic isolation and mtDNA introgression between *Ixodes persulcatus* and *I. pavlovskyi* [40] and between *I. ricinus* and *I. persulcatus*, was shown using both nuclear and mitochondrial DNA markers [41]. Natural hybridization was also observed in the African species *Hyalloma*, *H*. *dromedarii*, *H. truncatum*, and *H. marginatum rufipes*, with 10–14% mtDNA *COI* diversity. The mtDNA *COI* phylogeny revealed that multiple individuals of *H. dromedarii* and *H. truncatum* clustered together with the same mtDNA lineage as *H. marginatum rufipes*, indicating discordance between morphology and mtDNA phylogeny. On the basis of mtDNA, individuals with the morphology of *H. dromedarii* and *H. truncatum* are indistinguishable from *H. marginatum rufipes* [18]. Therefore, the presence of *H. scupense* in Clade III might be due to the cryptic hybridization with *H. impeltatum*, but an analysis of a nuclear DNA marker together with mtDNA is needed to provide evidence for this phenomenon. Moreover, AlKulabiyah (MK305816.1) and Hofuf (MH590864.1), haplotypes from camel ticks from eastern Saudi Arabia that were reported in Omer et al. [23] and included in the sequence data set, were clustered with *H. dromedarii*. The phylogenetic analysis of the populations of camel ticks from different geographic regions provides evidence of the existence of tree hard tick species in Ha’il Province. For example, a comprehensive survey on tick species composition collected from 18 domestic and wild animals in Riyadh province, Saudi Arabia, reported nine tick species from the bodies of camels, including the most dominant *H. dromedarii* and the fourth most common *H. impeltatum* [14].

As shown in Figure 4, mtDNA *COI* sequences from the tick samples infesting cattle, horses, sheep, and hedgehogs showed that these belong to *H. scupense*. These results clearly showed that these ticks that infest camels are differentiated from those infecting other mammals, indicating the presence of specific lineages infesting other mammals and host specificity. The most striking conclusion that could be inferred from these results is that the host specificity of ticks infesting other mammalian animals is genetically close and differentiated from those infesting camels. Earlier investigations suggested a correlation between modifications in mouthparts and coaxae in ticks, which are associated with host specificity due to their coevolution (e.g., [42,43,44]). On the other hand, major experimental studies put forward that ticks can feed and reproduce in a wide variety of hosts [42,44,45]. In the case of this study, a more comprehensive survey on tick host specificity in Saudi Arabia, covering more potential hosts, would address the range of the host specificity of ticks. The findings of such a survey are essential in managing and controlling tick-associated infections.

## 5. Conclusions

The findings of this study based on the barcoding mtDNA *COI* gene sequence analysis of nine haplotypes revealed the existence of two species in Ha’il province, namely, *H. dromedarii* and *H. impeltatum*. The phylogenetic analysis of these haplotypes combined with the previously reported ones have displayed two distinct clades: one aligned with *H. dromedarii* and the other aligned with *H. impeltatum*. The presence of the *H. scupense* and *H. impeltatum* clustering in the same clade suggests possible introgressive hybridization between these species. *H. scupense* infesting mammals other than camels indicates the existence of different lineages of this species, showing different host specificities.

## Figures and Tables

**Table 1:** Results of sequence analyses of mtDNA *COI* of the hard tick samples from the Ha’il region.

Number of Sites	500
Number of haplotypes, h:	9
Haplotype (gene) diversity, Hd:	0.657 ± 0.063
The variance of haplotype diversity:	0.00402
Nucleotide diversity, Pi	0.04954

**Table 2:** Haplotypes determined from the Ha’il region and their occurrence among the sampling locations. The haplotypes described for the first time in this study were written in bold.

	Al Qaid	Al Khotha	Al Ajfar
**H1-Ha’il-KSA**	14	9	5
**H2-Ha’il-KSA**	1	1	-
**H3-Ha’il-KSA**	1	-	-
**H4-Ha’il-KSA**	1	-	-
H5-Ha’il-KSA	1	-	-
H6-Ha’il-KSA	-	1	-
**H7-Ha’il-KSA**	-	2	-
**H8-Ha’il-KSA**	-	1	-
H9-Ha’il-KSA	-	-	13

**Table 3:** Results of analyses of the mtDNA *COI* sequences from the hard tick samples from different locations.

	Nucleotide Diversity, Pi(t)	Haplotype Diversity, Hd (SD)	Average Number of Nucleotide Differences, k
Al Qaid/Al Khotha	0.01031	0.505 ± 0.158	5.302
Al Qaid/Al Ajfar	0.05755	0.505 ± 0.158	40.938
Al Khotha/Al Ajfar	0.05974	0.628 ± 0.143	39.144

**Table 4:** Estimates of evolutionary divergence between the mtDNA *COI* haplotypes in the hard ticks from camels. The haplotypes in bold are identical based on the *COI* sequences obtained from this study.

	Haplotypes	1	2	3	4	5	6	7	8	9	10	11	12	13	14
**1**	**H1-AlKulabiyah KSA_MK305816.1**														
**2**	**H1-Ha’il-KSA**	0.000													
**3**	H2-Ha’il-KSA	0.002	0.002												
**4**	H3-Ha’il-KSA	0.004	0.004	0.004											
**5**	H4-Ha’il-KSA	0.008	0.008	0.010	0.012										
**6**	H7-Ha’il-KSA	0.128	0.128	0.131	0.133	0.133									
**7**	H8-Ha’il-KSA	0.131	0.131	0.134	0.136	0.136	0.002								
**8**	**H1-Hdro KSA_MK177460.1**	0.000	0.000	0.004	0.004	0.004	0.123	0.128							
**9**	H5-Hdro KSA MK177459.1	0.128	0.128	0.132	0.132	0.123	0.011	0.007	0.128						
**10**	H4-Hdro KSA MK177458.1	0.132	0.132	0.137	0.136	0.128	0.007	0.004	0.132	0.007					
**11**	H2-Hdro KSA MK177457.1	0.004	0.004	0.007	0.007	0.007	0.123	0.128	0.004	0.128	0.132				
**12**	**H1-*H. dromedarii* Ethiopia AJ437061.1**	0.000	0.000	0.002	0.004	0.008	0.128	0.131	0.000	0.128	0.132	0.004			
**13**	H3-*H. dromedarii* Australia AF132822.1	0.004	0.004	0.006	0.008	0.012	0.128	0.131	0.004	0.132	0.137	0.007	0.004		
**14**	**H2-*H. dromedarii* Iran KT920181.1**	0.000	0.000	0.002	0.004	0.008	0.128	0.131	0.000	0.128	0.132	0.004	0.000	0.004	
**15**	H5-*H. dromedarii* UAE MG188800.1	0.004	0.004	0.006	0.008	0.012	0.128	0.131	0.007	0.127	0.132	0.011	0.004	0.008	0.004

## Data Availability

All data are presented in the manuscript.
